# Relationship between Body Mass Index and Health-Related Physical Fitness Components in HIV-Diagnosed Children and Adolescents

**DOI:** 10.3390/children11080938

**Published:** 2024-08-02

**Authors:** João Antônio Chula de Castro, Luiz Rodrigo Augustemak de Lima, Diego Augusto Santos Silva

**Affiliations:** 1 Graduate Program of Physical Education, Sports Center, Federal University of Santa Catarina, P.O. Box 476, Florianopolis 88040-900, SC, Brazil; joaoantoniochula@gmail.com; 2 Institute of Physical Education and Sport, Federal University of Alagoas, Maceio 57072-900, AL, Brazil; luiz.lima@iefe.ufal.br

**Keywords:** acquired immunodeficiency syndrome, chronic disease, youth

## Abstract

Background/Objectives: There is a need to monitor physical fitness in HIV-diagnosed children and adolescents, and body mass index (BMI) could be an option for this due to its usability for assessing nutritional status and fat mass. The present study aimed to explore the relationship between BMI and physical fitness in HIV-diagnosed children and adolescents. Methods: A cross-sectional study was conducted with 86 HIV-diagnosed children and adolescents aged 5–15, with participants from two research protocols (Study I, *n* = 65; Study II, *n* = 21). Physical fitness was assessed through body composition (anthropometric measurements and dual energy X-ray absorptiometry), cardiorespiratory fitness (peak oxygen consumption [VO_2_peak]), muscle strength/endurance (handgrip strength, standing broad jump, and abdominal and modified push-up endurance), and flexibility (sit-to reach test). The relationship between BMI and physical fitness components was analyzed through correlation and simple and multiple linear regression analysis. Results: Eutrophic participants (mean age 11.44 ± 2.20) presented a normal fat mass percentage and overweight participants (mean age 11.50 ± 2.54) presented adequate handgrip strength. The adjusted models could explain 71% of fat-free mass, 57% of fat mass percentage, 70% of bone mineral content, 72% of bone mineral density, and 52% of handgrip strength. Conclusions: Increases in BMI were associated with increases in fat-free mass, fat mass percentage, bone mineral content, bone mineral density, and handgrip strength. BMI was capable of distinguishing those presenting a normal fat mass percentage and those presenting adequate handgrip strength.

## 1. Introduction

The investigation of health-related physical fitness in HIV-diagnosed children and adolescents has shown that they present with alterations in body composition, such as fat mass accumulation/distribution [1,2] and low bone development [3,4,5], and lower cardiorespiratory fitness, muscle strength/endurance, and flexibility, when compared to healthy populations [6,7]. This investigation has been led by the possible relation between these alterations and HIV infection status, as well as the adverse effects of the combination of drugs to suppress HIV replication, known as combined antiretroviral therapy (ART) [6,7,8,9,10,11,12,13,14,15,16]. However, different methods and protocols such as laboratory and field tests, and cut-points such as internationals reference values [16,17,18,19] and empirical cut-points [5,16,20,21,22,23] have been applied to investigate health-related physical fitness components in HIV-diagnosed children and adolescents [16]. Thus, this lack of standardization limits the comparison between studies’ results and the direction of guidelines for monitoring physical fitness in the clinical context [16,24,25,26,27,28].

Although previous studies described that alterations in health-related physical fitness in HIV-diagnosed children and adolescents can be related to fat mass accumulation [6,7], there are facts that suggest that the assessment of fat mass discriminators, such as body mass index (BMI), could be an alternative for monitoring physical fitness in HIV-diagnosed children and adolescents. BMI has been extensively investigated since the inception of research in this area [1,2,3], with an initial focus on monitoring growth development and stunting in HIV-diagnosed children and adolescents [16,29,30,31], and recently in research that aimed to evaluate the safety of ART-related medications and the potential effects of HIV infection and ART use [8,9,10,11], due to BMI’s usability for monitoring nutritional status and fat mass [27,28,32]. Additionally, BMI has been the primary measure recommended for monitoring body composition in HIV-diagnosed children and adolescents [25,26,27,28], and is also the most commonly used in studies assessing physical fitness in this population [16]. The emphasis on monitoring BMI in HIV-diagnosed children and adolescents, and its extensive investigation, is based on the observed relationship between growth parameters alterations (monitored via BMI) and changes in fat-free mass and bone mass [5,20,21,22,23] that can negatively impact muscle strength/endurance and cardiorespiratory fitness [24,25,26,27,28]. However, previous studies investigating HIV-diagnosed children and adolescents has focused mainly on describing BMI in this population and applying BMI as a control parameter in intervention studies [8,9,10,11,16]. Thus, less is known regarding the direct relationship between BMI and physical fitness components such as cardiorespiratory fitness and muscle strength/endurance and flexibility, as well as whether changes in BMI are associated with alterations in these components.

Considering the need for monitoring physical fitness in HIV-diagnosed children and adolescents, the broad investigation of BMI in this population, and its possible relationship with physical fitness components, the present study aimed to explore the relationship between BMI and health-related physical fitness components in this population.

## 2. Materials and Methods

### 2.1. Study Design and Sample

A cross-sectional study was conducted with 86 HIV-diagnosed children and adolescents, aged 5–15. The study sample was composed of participants from two research protocols, ‘Saúde PositHIVa Study’, with a data collection period between November 2015 and September 2016 (Study I, *n* = 65), and ‘Health-related Physical Fitness Assessment Guide for Children and Adolescents diagnosed with HIV infection’, with a data collection period between March 2022 and March 2023 (Study II, *n* = 21), developed in Florianopolis, Brazil, with children and adolescents undergoing clinical follow-up at the Joana de Gusmão Children’s Hospital at the time of the studies. The inclusion criteria were as follows: (i) medical records documenting confirmed HIV infection through vertical transmission, ART scheme, viral load, and CD4+ and CD8+ lymphocytes cell count (CD4; CD8); (ii) ability to stand or move; (iii) fully developed speech and/or no hearing impairment; (iv) absence of diseases that could alter body composition or were unrelated to HIV (e.g., hepatic insufficiency), and no medical issues contraindicating physical activity or affecting motor control pattern (e.g., advanced immunodeficiency in the presence of opportunistic infection); (v) non-continuous use of diuretic medication during the data collection period.

Power sample size was calculated through G*Power software version 3.1.9.7 (Heinrich-Heine-Universität Düsseldorf, Düsseldorf, Germany) with linear multiple regression and a posteriori parameters (one for tails, 0 for H0 ρ^2^, and 0.05 for “α err prob”), resulting in a power (1^−^ β err prob.) of 0.999 for calculated H1 ρ^2^ of 0.31 and 0.466, from an observed R^2^ of 0.31 and 0.50, with one or four number of predictors, respectively, and 86 for total sample size [33,34].

### 2.2. Participants’ Characteristics

The participants’ characteristics of sex and age were assessed through questionnaire, and the pubertal stage was classified through self-assessment based on the secondary sexual characteristics of development defined by Tanner [35]. Characteristics related to HIV infection were obtained from medical records: viral load (copies/mL), CD4+ lymphocytes cell count (cell mm^−3^) (CD4), and CD8+ lymphocytes cell count (cell mm^−3^) (CD8), measured through flow cytometry and RNA quantification, and the use of ART and type of ART (with or without protease inhibitor). Viral load was classified as follows: target not detected (≤20 copies/mL) or lower than detectable limit (≤40 copies/mL) and detectable (>40 copies/mL) [25,28,36]. The CD4 percentage (%CD4) was calculated through the CD4 cell count, which was as follows: >25% (>500 cell mm^−3^), 14–25% (200–499 cell mm^−3^), and <4% (<200 cell mm^−3^) [25,28,36]. The immunosuppression status was categorized using %CD4, according to the Center for Disease Control (CDC) parameters: Stage 1, non-immunosuppressed (>25% CD4); Stage 2, moderate immunosuppression (15–25% CD4); Stage 3, severe immunosuppression (<15% CD4) [36].

Considering that the physical activity level can promote improvements in health-related physical fitness components and can moderate the relationship between BMI and different components [24,37], the physical activity level was investigated using the Portuguese version of the Physical Activity Questionnaire for Older Children (PAQ-C) [38], in which physical activity is assessed through nine items related to daily activities in three contexts (sports, leisure, and physical education) [39], and the answers are scored from one to five and grouped to calculate the average PAQ-C score to estimate the physical activity level [39]. The PAQ-C was previously reported with an adequate reliability (interclass correlation coefficient: 0.75 and 0.82 for male and female youths, respectively) [40] and validated for investigating the physical activity level in HIV-diagnosed children and adolescents (correlation coefficient: 0.506, sensitivity: 0.625, and specificity: 0.875) [41].

### 2.3. Health-Related Physical Fitness

Body composition was assessed through anthropometric measurements (height [cm] and body mass [kg]), dual energy X-ray absorptiometry, a whole-body composition scan (fat-free mass [kg], fat mass [kg], bone mineral content [BMC] [g] and bone mineral density [BMD] [g/cm^2^]). An AlturaExata^®^ stadiometer (Belo Horizonte, Brazil) and a digital scale Tanita^®^ BF683W (Arlington Heights, IL, USA) were used to measure the height and body mass, respectively, and participants were oriented to be wearing light clothes and be barefoot. Participants’ weight status was classified through BMI-for-age z-scores, after the body mass index (BMI) had been calculated through body mass and height (BMI *=* body mass/height^2^) in kg/m^2^, according to the World Health Organization (WHO) growth charts in which the following cut-off values were applied: severe thinness: <−3SD; thinness: <−2SD; overweight: >+1SD; obesity: >+2SD, and eutrophic: ≥−2SD and ≤+1SD [42,43]. Fat-free mass, fat mass, BMC, and BMD were evaluated through the DXA equipment GE^®^ Lunar Prodigy Advance and EnCore 2004 software version 8.10.027 (Study I) (GE Lunar Corporation, Madison, WI, USA) and Hologic^®^ Discovery Wi Fan-Beam-S/N 81593, (HOLOGIC, Inc., Bedford, MA, EUA) with pediatric software Hologic Auto Whole-Body version 12.4:5 (Study II). Daily and weekly calibrations following manufacture recommendations were performed. Participants were instructed to be barefoot, wear light clothes and not to wear any kind of metal adornments.

Cardiorespiratory fitness was investigated through peak oxygen consumption (VO_2peak_) in ml.kg-1.min-1 and peak heart rate in beats per minute (bpm); these were assessed using an incremental cycle ergometer test with a breath-by-breath respiratory exchange evaluation, following a previously established protocol [44]. In Study I, the cycle ergometer Ergofit^®^ 167 (Ergofit^®^, Toledo, Spain) and the gas analyzer K4bs (COSMED, Rome, Italy) were used. In, Study II, the cycle ergometer Lode Excalibur Sport (Lode BC, Groningen, The Netherlands) and the gas analyzer Quark CPET (COSMED, Rome, Italy) was used. Daily calibrations were performed in both studies, following manufacture recommendations, and the raw data were smoothed (3s). The he cardiac monitor Polar^®^ S610i, with a heart rate chest strap sensor (Polar^®^ electro, Oy, Kempele, Finland), was used to perform the tests in both studies. The cycle ergometer test protocol consisted of a pre-test (five-minute rest parameter measurement), warm-up (three-minute cycling with a load of 20 watts and cadence of 50–60 rotations per minute [rpm]), rest interval (two-minute rest parameters measurement), exercise baseline (five-minute cycling, with 20 watts of load and 50–60 rpm of cadence), and maximum effort exercise (with 50–60 rpm of constant cadence, load starting in 20 watts and increased by 15 watts every minute [one watt each four seconds] until voluntary exhaustion). Participants were verbally encouraged to perform the maximal effort; the parameters of the respiratory exchange ratio (also, respiratory quotient) ≥ 1.1, the effort perception on the Borg scale >17, and the inability to maintain the cadence between 50 and 60 rpm were applied.

Muscle strength/endurance was investigated through hand grip strength (kg), standing broad jump distance (cm), and an abdominal and modified push-up endurance test (number of repetitions in one minute [reps/min]). The hydraulic handgrip dynamometer Saehan^®^ SH5001 (Saehan Corporation, Masan, Republic of Korea) was used to assess the handgrip strength in both studies, in which participants were oriented to be in a stand position with elbows extended [45]. Two measurements of each limb were assessed, alternating the evaluated limb, starting with the right limb alternating to the left limb, and shortly thereafter measuring again the right limb alternating to the left limb. The sum of the higher value for each limb considered the statistical analysis. The standing broad jump was used to estimate the lower limbs strength in Study II, in which participants were oriented to jump as far as they could, using their arms as best fitted. Two attempts were performed, and the longest jump considered the statistical analysis [46]. The abdominal resistance test was used in both studies to estimate the abdominal muscle endurance in which the maximum number of complete repetitions in one minute, or up to 75 repetitions, was considered [47]. The upper limbs’ muscle endurance was estimated through the modified push-up test in Study II, in which the maximum number of repetitions in one minute was considered [45].

Flexibility was evaluated in Study II through the sit-to reach test with a Wells bench test (Sanny^®^, São Bernardo do Campo, Brazil) in which three attempts were performed, and the higher value was considered for statistical analysis [45].

The physical fitness components protocol assessment started with the evaluation of muscle strength/endurance (handgrip strength, stand broad jump, modified push-ups, and abdominal endurance), followed by the flexibility evaluation; for each muscle that underwent a strength/endurance test and flexibility test, a familiarization period was applied for movement/posture adjustments and there was also a brief warm-up. The cardiorespiratory fitness assessment was carried out during a second visit to avoid fatigue from previous tests [45,46,47]. Physical fitness components were classified using reference values previous published and applied to evaluate HIV-diagnosed children and adolescents [16], in which body fat percentage was classified as normal or high [48], BMD as normal or low [49], and the VO_2peak_ [50], handgrip strength [51], standing broad jump [51], abdominal endurance [48], modified push-ups [52], and sit-to-reach flexibility [48] were classified as adequate or low.

### 2.4. Statistical Analysis

The continuous variables were presented as mean and standard deviation, and the categorical variables as frequencies and percentages to the descriptive analysis. Data normal distribution was evaluated through a Shapiro–Wilk test, histograms, and scatter plots, comparing the study data to a theoretical normal distribution [53]. Sexes and groups differences (Study I and Study II; eutrophic and overweigh participants) were assessed using an independent samples *t*-test (normally distributed continuous data) or a Mann–Whitney U Ranked Sum test (non-normally distributed continuous data), or a Pearson’s chi-squared test (categorical data). The relationship between BMI and health-related components was initially analyzed through Spearman correlation tests and graphical analysis. Simple and multiple linear regression analyses were performed to investigate the direct association between the dependent variable (physical fitness components) and the independent variable BMI (simple regression) and whether this association was maintained after the adjustment of the models (multiple regression). The models were initially adjusted through a backwards method, considering possible differences between the participants of both studies (group: Study I and II) and previously established associations between the components of physical fitness, and/or BMI, with the variables of age, sex, pubertal stage and level of physical activity [16,24,37,43,49,51,52,54]. Moreover, the models were evaluated through significance (*p*-value < 0.05), standard error of estimate, residual normality analysis (Shapiro–Wilk test), Akaike’s Inflation Criteria, and the Bayesian Information Criterion. Additionally, to avoid multicollinearity, model overfitting, or a lack of statistical significance, independent variables with a correlation coefficient ≥ 0.75 between them were not included in the same model, and variables that resulted in a Variance Inflation Factor > 5 were removed from the models, as well as those without statistical significance that did not generate adjustments in the models [55]. The R© 4.2.1 (The R Foundation for Statistical Computing, Vienna, Austria) software and packages were used to perform all statistical analyses, and missing data were excluded from the analysis.

## 3. Results

### 3.1. Participants’ Characteristics

Table 1 presents the participants’ characteristics of the total of 86 children and adolescents who met the inclusion criteria. Besides the different inclusion criteria between both studies regarding age, there was no significant difference between participants of both studies regarding mean age, BMI, and the percentage of females and males in each study. However, the participants of Study I presented a higher BMC, BMD, and VO_2peak_ when compared to participants of Study II, and the participants of Study II presented a higher fat mass percentage and peak heart rate when compared to participants of Study I. The participants of Study II presented a higher CD4/CD8 ratio when compared to participants of Study I. Study I presented a higher percentage of participants with detectable viral load, participants treated with ART with protease inhibitors, and participants not treated with ART. Moreover, one participant presented obesity and two participants presented thinness; results that only allowed a comparison between eutrophic and overweight participants considering those BMI categories (obesity and thinness) did not present a sufficient sample.

When the difference between the eutrophic and overweight participants is observed, eutrophic participants presented a higher VO_2peak_ and modified push-ups values when compared to overweight participants, and overweight participants presented a higher fat-free mass, fat mass, and fat mass percentage when compared to eutrophic participants (Table 2). Moreover, eutrophic participants presented normal fat mass percentage and low handgrip strength, and overweight participants presented high fat mass percentage and adequate handgrip strength (Table 3).

### 3.2. Association between BMI and Physical Fitness Components (Correlation, Simple and Multilinear Regression Analysis

**Figure 1:**
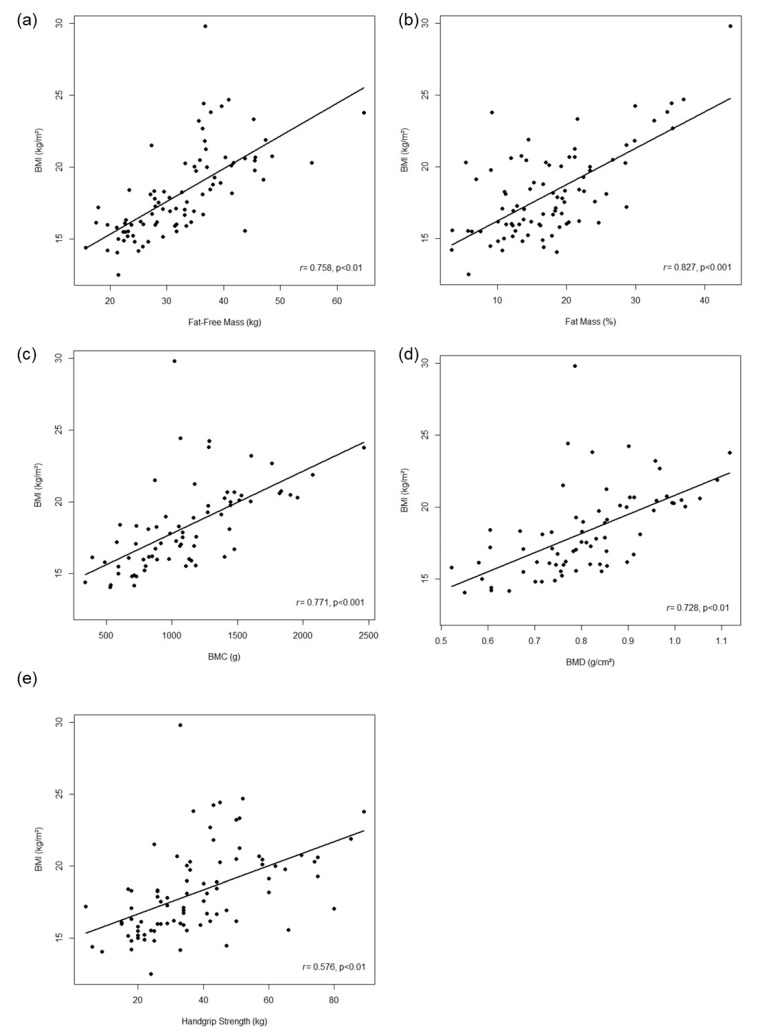
Correlation between BMI and health-related physical fitness components, scatterplot analysis. BMI: body mass index; BMC: bone mineral content; BMD: bone mineral density. (**a**) fat-free mass; (**b**) fat mass percentage; (**c**) bone mineral content; (**d**) bone mineral density; (**e**) handgrip strength.

A significant positive correlation was observed between BMI and fat-free mass, fat mass percentage, BMC, BMD, and handgrip strength, in which the values for BMI were higher than the values for fat-free mass, fat mass percentage, BMC, BMD, and handgrip strength (Table 4, Figure 1). A similar result was observed in the linear regression analysis, in which BMI could explain 44% of fat-free mass, 43% of fat mass percentage, 37% of BMC, 33% BMD, and 24% of handgrip strength, and models adjusted by age, sex and group could explain 71% of fat-free mass, 57% of fat mass percentage, 70% of BMC, 72% of BMD, and 52% of handgrip strength. Regarding the VO_2peak_, no significant correlation was observed between the BMI and VO_2peak_, and BMI could only explain 12% of the VO_2peak_ in the linear regression analysis and in the adjusted models; despite its significance, the standardize coefficient was only −0.03 and the model explained 33% of VO_2peak_. There was no significant association between BMI and the standing broad jump, as well as with abdominal endurance (Table 5). The models were adjusted initially by age, sex, group (Study I and II), pubertal stage, and physical activity level. However, the pubertal stage and physical activity level correlated highly with the variable sex (rho > 0.75) and did not present significance in models. Thus, those variables were removed from the final adjusted models to avoid overfitted models and non-significant model adjustments.

## 4. Discussion

This study investigated the relationship between BMI and different health-related physical fitness components in HIV-diagnosed children and adolescents, and the main finds were the direct association between BMI and fat mass percentage, fat-free mass, BMC, BMD, and handgrip strength. Additionally, BMI status was associated with changes in fat mass percentage and handgrip strength.

The relationship between BMI and body composition components such as fat mass percentage is one of the principles applied to the development of the WHO growth charts, due to the usability of BMI to identify those with a higher body fat mass and fat mass percentage [42,54]. Thus, the association between BMI and fat mass and BMI and fat mass percentage was an expected result, much like the association between BMI classified according to the WHO growth charts and fat mass percentage classification (normal and high fat mass percentage). However, BMI was also positively associated with the fat-free mass results that corroborate previous studies, which showed that BMI was not capable of distinguishing between fat mass and fat-free mass [49,56]. Thus, the results showed that BMI can be applied to identify those with a higher fat mass percentage with a limitation that will not distinguish those with higher BMI values due to higher fat-free mass.

The results regarding BMC and BMD agree with previous studies that investigated bone mass in HIV-diagnosed children and adolescents, showing that increases in BMI are related to an increase in BMC and BMD [9,57,58,59]. The association between BMI and BMC and BMI and BMD can be explained by the fact that BMI can be increased due to a greater fat-free mass [49,56], and the recruitment of mineral cells to improve bone structure is related to the mechanical loading applied to bones that in turn is related to the capacity of muscle force production [60]. These relationships were shown in the present study when the positive association between BMI and fat-free mass was observed, as well as the positive correlation between fat-free mass, BMC, and BMD. However, BMI classified with the WHO growth charts was not capable of distinguishing greater values of BMC and BMD between eutrophic and overweight participants, as well as distinguishing participants with adequate and low BMD. Considering that HIV-diagnosed children and adolescents can present alterations in bone development [9,57,58,59], bone mass parameters should be monitored in this population, and should be BMI the index present in recommendations for monitoring this population [25,26,27,28]. The results indicated that, besides the association with BMC and BMD, BMI was not an adequate parameter to distinguish between those with lower BMD and those with normal BMD.

Regarding muscle strength/endurance, there is no sufficient evidence regarding the association between BMI and muscle strength/endurance in HIV-diagnosed children and adolescents due to lack of studies [16]. Moreover, previous studies had shown no difference in muscles strength/endurance [14], lower muscles strength/endurance [7] or high muscles strength/endurance [61] when comparing HIV-diagnosed children with their healthy peers. In the present study, BMI was directly associated with handgrip strength and changes in BMI status was associated with changes in handgrip strength. As previously described, increases in BMI can be related to increases in fat-free mass [49,56] and this can be the explanation for the relationship between BMI and muscle strength/endurance. Moreover, previous studies described that BMI could negative affect muscle strength/endurance in tests involving body movement and positive affect isometric tests, since higher values for BMI can also be related with higher fat mass and fat-free mass [62,63]. The results from the present study regarding handgrip strength agree with those hypotheses. However, when observed the results regarding standing broad jump, abdominal endurance, and modified push-ups, BMI itself was not directly associated with results from tests involving body movement. The results from the present study could be explained by the fact that overweight participants, besides presenting higher fat mass and fat mass percentage, presented higher fat-free mass when compared with eutrophic participants. Conversely, higher values of standing broad jump, abdominal endurance and modified push-ups were not related to fat-free mass, but correlated with fat mass percentage, and adjusted models were significant for stand broad jump and abdominal endurance, results that indicated that variables such as age, sex and fat mass percentage could be more useful than BMI to investigate standing broad jump, abdominal endurance and modified push-ups.

Concerning to VO_2peak_, significant association between BMI and VO_2peak_ was observed in linear regression analysis, but in the adjusted model BMI standardized coefficient was only −0.03, suggesting that despites its significant association, BMI contribute little to the model estimates. Furthermore, alterations in BMI status were not associated with alterations in VO_2peak_. However, a significant correlation between VO_2peak_ and fat mass percentage was observed. This result agreed with previous studies that investigate cardiorespiratory fitness in HIV-diagnosed children and adolescents and reported low VO_2peak_ associated with high fat mass percentage [6,18]. However, those studies did not investigate if this association was also applied to fat mass discriminators such as BMI. The investigation of the association between VO_2peak_ and BMI considered previous studies that described the association between high cardiorespiratory fitness and normal weight status expressed by BMI and attributed this relationship to the fact that overweight and obese individuals present higher fat mass and fat mass percentage and thus presented lower cardiorespiratory fitness [64,65]. However, considering the present study results BMI was not an adequate alternative for monitoring VO_2peak_ alterations in HIV-diagnosed children and adolescents. Thus, variables such as age, sex and fat mass percentage could be more useful compared to BMI to investigate VO_2peak_ in this population.

Regarding flexibility, previous studies described a lower flexibility for HIV-diagnosed children and adolescents compared to their healthy peers, which could be related to a decreased physical activity level [6,7,15]. However, those studies only described results regarding flexibility and did not investigate associations between flexibility and an anthropometric index such as BMI [6,7,15]. Moreover, results from studies investigating exercise interventions in HIV-diagnosed populations are inconsistent with results describing an improvement in flexibility [66] and results describing a decrease in flexibility [13] after the intervention. Thus, previous finds regarding flexibility are not clear [16]. The results from the present study agree with previous studies describing low flexibility in HIV-diagnosed children and adolescents [6,7,15]. However, BMI was not associated with flexibility, and flexibility correlated only with the standing broad jump, in which higher values for flexibility correlated with higher values for standing broad jump. The lack of association between BMI and flexibility was previous described, as well as the association between BMI and flexibility through secondary factors such as muscular tension; results that sustain this hypothesis suggest that flexibility should not be investigated alone but in combination with other musculoskeletal variables [67]. This hypothesis is based on the definition of flexibility being the range of motion of a muscle and connective tissues at a joint or group of joints; thus, the direct associations regarding flexibility may not be clear, with flexibility potentially impacting results of other variables such as the standing broad jump [67]. Thus, the results from the present study confirmed that flexibility can be related to results for other musculoskeletal variables.

Despite the results, the present study has limitations, such as the small number of participants presenting with thinness and obesity not allowing us to hypothesize whether the observed relationship between BMI and health-related physical fitness for those in the eutrophic and overweight categories could be the same. Moreover, the statistical analysis that was performed grouped participants from two studies whose protocols were performed during different periods, with the COVID-19 pandemic happening in the gap between the studies; this could generate differences between the groups regarding behavior variables such as physical activity. However, the adoption of this strategy was continued, considering that no differences between the participants from both studies were observed regarding the sex distribution, age, and BMI, as well as the physical activity level; the models were adjusted considering groups.

## 5. Conclusions

Through the results of the present study, it was concluded that increases in BMI were associated with increases in fat-free mass, fat mass percentage, BMC, BMD, and handgrip strength. Moreover, alterations in BMI were associated with alterations in the fat mass percentage and handgrip strength. Thus, BMI could be an alternative anthropometric index for monitoring alterations in fat mass, handgrip strength, and BMC and BMD, in which high values of BMI were associated with high fat mass percentage, and low values of BMI were associated with a low handgrip strength and low values of BMC and BMD in HIV-diagnosed children and adolescents.

## Figures and Tables

**Table 1:** Study participants’ characteristics.

Variables	Study I	Study II	Total
	(*n* = 65) (75.6%)	(*n* = 21) (24.4%)	(*n* = 86)
	Mean (SD)	Mean (SD)	Mean (SD)
Age (years)	11.71 (2.08)	10.62 (2.42)	11.44 (2.20)
Height (cm)	147.34 (13.08)	143.18 (13.28)	146.32 (13.17)
Body mass (kg)	39.85 (11.37)	40.05 (13.75)	39.90 (11.91)
BMI (kg/m^2^)	17.94 (2.66)	18.97 (4.05)	18.19 (3.06)
Fat-free mass (kg)	32.95 (9.31)	30.90 (8.41)	32.44 (9.09)
Fat mass (kg)	6.91 (4.34)	9.19 (6.99)	7.47 (5.17)
Fat mass (%)	16.73 (7.18)	**20.70 (9.84)** *	17.71 (8.05)
BMC (g)	**1182.93 (445.69)** *	938.02 (337.55)	1109.46 (428.94)
BMD (g/cm^2^)	**0.84 (0.12)** *	0.74 (0.14)	0.81 (0.13)
VO_2_peak (mL∙kg^−1^∙min^−1^)	**39.11 (6.86)** *	33.86 (8.87)	37.97 (7.60)
Peak heart rate (bpm)	168.70 (14.57)	**176.89 (12.66)** *	170.59 (14.50)
Handgrip strength (kg)	39.92 (19.14)	31.57 (14.19)	37.88 (18.34)
Stand broad jump (cm)	NI	116.92 (31.07)	-
Abdominal endurance (reps/min)	17.65 (14.68)	27.19 (10.70)	19.98 (14.36)
Modified push-ups (reps/min)	NI	24.62 (10.05)	-
Sit-to-reach flexibility (cm)	NI	23.02 (5.20)	-
PAQ-c score	2.44 (0.77)	2.57 (0.79)	2.47 (0.77)
CD4 count (cells/uL)	857.63 (367.73)	1020.67 (410.28)	897.44 (382.61)
CD8 count (cells/uL)	1185.11 (547.86)	1039.33 (427.94)	1149.51 (522.54)
CD4/CD8 ratio	0.84 (0.42)	**1.06 (0.41)** *	0.89 (0.43)
Time of ART (years)	-	7.81 (4.56)	-
Sex	*n* (%)	*n* (%)	*n* (%)
Females	35 (53.8)	11 (52.4)	46 (53.5)
Males	30 (46.2)	10 (47.6)	40 (46.5)
BMI (WHO grow charts)			
Thinness	2 (3.1)	0 (0.0)	2 (2.3)
Eutrophic	55 (84.6)	16 (76.2)	71 (82.6)
Overweight	8 (12.3)	4 (19.0)	12 (13.9)
Obesity	0 (0.0)	1 (4.8)	1 (1.2)
Physical activity level			
Met PA guidelines	36 (55.4)	16 (76.2)	52 (60.5)
Did not meet PA guidelines	29 (44.6)	5 (23.8)	34 (39.5)
Viral load (copies/mL)			
TND or LDL (≤20 or ≤40)	44 (67.7%)	20 (95.2%)	64 (74.4%)
41–1000	**9 (13.8%)** +	1 (4.8%)	10 (11.6%)
>1000	**12 (18.5%)** +	0 (0.0%)	12 (14.0%)
CD4 count (cells/uL)			
<200	2 (3.1%)	0 (0.0%)	2 (2.3%)
200–499	7 (10.8%)	2 (9.5%)	9 (10.5%)
≥500	56 (86.2%)	19 (90.5%)	75 (87.2%)
ART use			
ART with PI	**39 (60.0%)** +	6 (28.6%)	45 (52.3%)
ART without PI	15 (23.1%)	15 (71.4%)	30 (34.9%)
Without ART	**11 (16.9%)** +	0 (0.0%)	11 (12.8%)

SD: standard deviation; BMI: body mass index; BMC: bone mineral content; BMD: bone mineral density; VO_2peak_: peak oxygen consumption; NI: not investigated; PAQ-C score: physical activity questionnaire for older children final score; CD4 count: CD4 lymphocytes cell count; CD8 count: CD8 lymphocytes cell count; ART: antiretroviral therapy; PA: physical activity; TND: target not detected; LDL: lower than detectable limit; PI: protease inhibitor; * independent variables *t*-test or Wilcoxon signed-rank test *p*-value < 0.05; ^+^ chi-squared test *p*-value < 0.05; significant differences are in bold.

**Table 2:** Difference between BMI groups (classified according to WHO growth charts) and participants’ characteristics.

Characteristic	BMI Eutrophic (*n* = 71) (85.5%)	BMI Overweight (*n* = 12) (14.5%)	*p*-Value ^a^
Age (years)	11.44 (2.20)	11.50 (2.54)	0.875
Height (cm)	145.39 (13.30)	152.32 (12.58)	0.096
Body mass (kg)	37.70 (10.11)	**52.76 (12.01)**	<0.01
BMI (kg/m^2^)	17.43 (2.13)	**22.39 (2.20)**	<0.01
Fat-free mass (kg)	31.49 (8.49)	**38.58 (10.93)**	0.012
Fat mass (kg)	6.19 (3.15)	**14.18 (5.98)**	<0.01
Fat mass (%)	16.09 (5.92)	**26.97 (8.76)**	<0.01
BMC (g)	1072.93 (393.18)	1358.91 (620.71)	0.066
BMD (g/cm^2^)	0.80 (0.13)	0.86 (0.17)	0.202
VO_2peak_ (mL.kg^−1^∙min^−1^)	**38.74 (7.09)**	32.55 (6.38)	0.001
Peak heart rate (bpm)	170.05 (14.87)	175.27 (12.72)	0.276
Handgrip strength (kg)	36.65 (17.85)	47.00 (21.19)	0.085
Stand broad jump (cm)	121.87 (33.11)	99.75 (19.87)	0.248
Abdominal endurance (reps/min)	20.31 (15.13)	18.58 (11.35)	0.928
Modified push-ups (reps/min)	**27.25 (8.83)**	14.25 (10.15)	0.019
Sit-to-reach flexibility (cm)	23.71 (5.03)	22.50 (4.88)	0.670
PAQ-c score	2.48 (0.77)	2.20 (0.74)	0.292
CD4 count (cells/uL)	899.89 (387.56)	827.50 (381.33)	0.400
CD8 count (cells/uL)	1151.82 (542.65)	1115.08 (456.62)	0.959
CD4/CD8 ratio	0.89 (0.41)	0.89 (0.57)	0.971
Time of ART (years)	7.69 (4.59)	7.50 (5.45)	0.944

BMI: body mass index; BMC: bone mineral content; BMD: bone mineral density; VO_2peak_: peak oxygen consumption; PAQ-C score: physical activity questionnaire for older children final score; CD4 count: CD4 lymphocytes cell count; CD8 count: CD8 lymphocytes cell count; ART: antiretroviral therapy; ^a^ independent variables *t*-test or Wilcoxon signed-rank test; significant differences are in bold.

**Table 3:** Association of BMI status and participants’ characteristics status, chi-square test.

Physical Fitness Component	BMI Eutrophic *n* (%)	BMI Overweight *n* (%)	Total *n*	X^2^	*df*
Fat mass (%)					
Normal	**67 (95.7)**	2 (16.7)	69	42.24	1
High	3 (4.3)	**10 (83.3)**	13		
BMD (g/cm^2^)					
Normal	47 (82.5)	0 (0.0)	47	0.58	1
Low	10 (17.5)	8 (100.0)	18		
VO_2peak_ (mL∙kg^−1^∙min^−1^)					
Adequate	40 (58.8)	3 (25.0)	43	3.43	1
Low	28 (41.2)	9 (75.0)	37		
Handgrip strength (kg)					
Adequate	18 (25.4)	**7 (58.3)**	25	3.85	1
Low	**53 (74.6)**	5 (41.7)	58		
Stand broad jump (cm)					
Adequate	5 (31.2)	0 (0.0)	5	0.42	1
Low	11 (68.8)	4 (100.0)	15		
Abdominal endurance (reps/min)					
Adequate	43 (60.6)	7 (58.3)	50	<0.01	1
Low	28 (39.4)	5 (41.7)	33		
Modified push-ups (reps/min)					
Adequate	15 (93.8)	3 (75.0)	19	0.03	1
Low	1 (6.2)	1 (25.0)	2		
Sit-to-reach flexibility (cm)					
Adequate	7 (43.8)	1 (25.0)	8	0.01	1
Low	9 (56.2)	3 (75.0)	13		

BMI = body mass index; X^2^: chi-square value; *df*: degrees of freedom; BMD: bone mineral density; VO_2peak_: peak oxygen consumption. Bold: *p*-value < 0.05.

**Table 4:** Spearman rank correlation between participants characteristics’ (BMI, health-related physical fitness components, age, and physical activity).

	BMI	FFM	FM%	BMC	BMD	VO_2peak_	HGS	SBJ	AbdE	MPU
BMI	1.000									
FFM	**0.758** **	1.000								
FM%	**0.827** ***	0.385	1.000							
BMC	**0.771** ***	**0.924** ***	0.443	1.000						
BMD	**0.728** **	**0.908** ***	0.360	**0.981** ***	1.000					
VO_2peak_	−0.424	−0.348	−**0.595** *	−0.453	−0.408	1.000				
HGS	**0.576** **	**0.711** **	0.237	**0.760** **	**0.785** ***	−0.190	1.000			
SBJ	−0.104	0.283	−**0.474** *	0.205	0.308	0.110	0.410	1.000		
AbdE	−0.128	0.085	−**0.510** *	−0.025	0.048	**0.726** **	0.314	0.407	1.000	
MPU	−0.304	−0.054	−**0.595** **	−0.162	−0.092	**0.513** *	0.215	**0.487** *	**0.824** ***	1.000
STRF	−0.308	0.004	−0.388	−0.152	−0.120	0.240	0.033	**0.467** *	0.183	0.018

BMI: body mass index; FFM: fat-free mass; FM%: fat mass percentage; BMC: bone mineral content; BMD: bone mineral density; VO_2peak_: peak oxygen consumption; HGS: handgrip strength; SBJ: standing broad jump; AbdE: abdominal endurance; MPU: modified push-ups; STRF: sit-to-reach flexibility; PA: physical activity. * *p* < 0.05; ** *p* < 0.01; *** *p* < 0.001; significant correlations are in bold.

**Table 5:** Association between BMI and health-related physical fitness components, simple and multilinear regression analysis.

	^β^ (95%CI)	^β^ *p*-Value	^β^ st	R^2^ Adjusted	*p*-Value	RMSE	F	VIF
Fat−free mass (kg)								
Simple regression	1.98 (1.51; 2.46)	**<0.001**	0.67	0.44	**<0.001**	6.78	68.28	
Multiple regression ^a^	1.34 (0.93; 1.75)	**<0.001**	0.45	0.71	**<0.001**	4.90	52.44	1.44
Fat mass (%)								
Simple regression	1.73 (1.30; 2.16)	**<0.001**	0.66	0.43	**<0.001**	6.07	64.46	
Multiple regression ^a^	1.81 (1.36; 2.25)	**<0.001**	0.69	0.57	**<0.001**	5.25	29.29	1.44
Bone mineral content (g)								
Simple regression	88.40 (61.27; 115.54)	**<0.001**	0.63	0.37	**<0.001**	339.30	42.26	
Multiple regression ^a^	58.63 (36.35; 80.90)	**<0.001**	0.42	0.70	**<0.001**	236.50	40.47	1.42
Bone mineral density (g/cm^2^)								
Simple regression	0.02 (0.02; 0.03)	**<0.001**	0.59	0.33	**<0.001**	0.11	34.44	
Multiple regression ^a^	0.02 (0.01; 0.02)	**<0.001**	0.38	0.72	**<0.001**	0.07	45.53	1.42
VO_2peak_ (mL∙kg^−1^∙min^−1^)								
Simple regression	−0.89 (−1.40; −0.38)	**0.001**	−0.36	0.12	**0.001**	7.13	12.18	
Multiple regression ^a^	−0.53 (−1.06; −0.01)	**0.047**	−0.03	0.33	**<0.001**	6.20	11.30	1.43
Handgrip strength (kg)								
Simple regression	2.99 (1.87; 4.12)	**<0.001**	0.01	0.24	**<0.001**	15.97	28.01	
Multiple regression ^a^	1.76 (0.70; 2.83)	**0.001**	0.29	0.52	**<0.001**	12.67	24.24	1.43
Stand broad jump (cm)								
Simple regression	−0.70 (−4.37; 2.97)	0.694	−0.07	−0.04	0.694	31.74	0.16	
Multiple regression ^a^	−1.89 (−5.37; 1.59)	0.267	−0.19	0.35	**0.015**	25.01	4.62	1.55
Abdominal endurance (reps/min)								
Simple regression	0.67 (−0.33; 1.68)	0.188	0.01	0.01	0.188	14.30	1.76	
Multiple regression ^a^	−0.22 (−1.29; 0.86)	0.688	−0.05	0.21	**<0.001**	12.80	6.50	1.43
Modified push-ups (reps/min)								
Simple regression	−0.79 (−1.92; 0.33)	0.158	−0.24	0.05	0.158	9.77	2.16	
Multiple regression ^a^	−0.96 (−2.22; 0.31)	0.129	−0.29	0.18	0.099	9.11	2.44	1.55
Sit-to-reach flexibility (cm)								
Simple regression	0.30 (−0.90; 0.29)	0.302	−0.18	0.01	0.302	5.18	1.13	
Multiple regression ^a^	−0.54 (−1.26; 0.18)	0.133	−0.32	0.01	0.411	5.19	1.01	1.55

BMI = body mass index; ᵝ: unstandardized regression coefficient for BMI; CI: confidence interval; ᵝ st: standard regression coefficient for BMI; R^2^ Adjusted: adjusted coefficient of determination; RMSE = root mean square error of estimate; F: F statistic; VIF = variance inflation factor; VO_2peak_: peak oxygen consumption; ^a^ models adjusted by age, sex and group. Bold: *p*-value < 0.05.

## Data Availability

The data presented in this study are available on request from the corresponding author due to ethical restrictions.
